# Green on Bronchitis

**Published:** 1847-04

**Authors:** 


*Green on Bronchitis.*—We observe in some of our Exchange Journals some bitterness of discussion respecting a new work by Dr. Green, of New-York City. It seems that Dr. G. recommends topical applications to the larynx by means of a sponge, which he has ascertained may be introduced with ease and safety down to the rima glottidis. Trousseau and Belloc, French authors, recommended this plan of treatment several years ago. The discussion relates to an undue assumption of originality by Dr. Green. When the work reaches this meridian we shall be better prepared to determine whether the charges against Dr. G. are just, or are the suggestions of that "green eyed monster that makes the food it feeds upon."

